# Are All Laws of Nature Created Equal? Meta-laws Versus More Necessary Laws

**DOI:** 10.1007/s10670-023-00739-7

**Published:** 2023-09-07

**Authors:** Salim Hirèche, Niels Linnemann, Robert Michels

**Affiliations:** 1 Department of Philosophy, University of Geneva, Geneva, Switzerland; 2 LanCog, Centre of Philosophy, University of Lisbon, Lisbon, Portugal

## Abstract

Two approaches to elevating certain laws of nature over others have come to prominence recently. On the one hand, according to the *meta-laws* approach, there are meta-laws, laws which relate to laws as those laws relate to particular facts. On the other hand, according to the *modal*, or *non-absolutist*, approach, some laws are necessary in a stricter sense than others. Both approaches play an important role in current research, questioning the ‘orthodoxy’ represented by the leading philosophical theories of natural laws—Humeanism, the DTA view, dispositional essentialism and primitivism. This paper clarifies the relations between these two emerging approaches, as well as their applicability to physical laws and the status of the challenges they pose for standard theories of laws of nature. We first argue that, despite some significant similarities between the two approaches (especially in the context of Lange’s counterfactual account of laws), they are in general distinct and largely independent of each other. Then, we argue that the support for meta-laws from physical theory and practice is more questionable than usually presented.

## Introduction

Are all laws of nature on a par? Some contributors to the recent literature have argued that this is not the case, since some laws are in a certain sense prior to others. There are two proposals for how this divide between two kinds of laws is drawn: According to the *meta-law approach*, we should distinguish between regular laws, laws which concern how things are in the world, and meta-laws, laws which concern other laws in the same manner as regular laws concern how things are in the world (see Lange, 2007; Yudell, 2013). Meta laws are prior to regular laws in the sense that they are ‘laws of laws [...] governing (and helping to explain) ordinary (i.e., non-meta, ‘first-order’) laws in a manner precisely analogous to the way in which those laws govern (and help to explain) ordinary facts and events.’ (Lange, 2007, p. 458).

Similarly, a number of authors have proposed *non-absolutist accounts* of the laws of nature, which claim that laws can have different modal statuses. More specifically, defenders of this modal approach to elevating certain laws of nature have argued that there is a division between laws which are merely nomically necessary and laws which are metaphysically necessary, i.e. necessary in a stricter, or perhaps the strictest sense. (See Hendry & Rowbottom, 2009; Tahko, 2015; Hirèche et al., 2021a; Hirèche et al., 2021b.) Laws in the latter category are prior to those in the former in virtue of this stricter modal status they possess.

Both approaches are relatively recent, each of them going against the orthodoxy in some important respect: none of the standard philosophical theories of laws—dispositional essentialism (Ellis, 2001; Bird, 2007), the DTA view (Dretske, 1977; Tooley, 1977; Armstrong, 1983), the Humean Best System Analysis (Lewis, 1973), or primitivism (Carroll, 1994; Maudlin, 2007; Chen & Goldstein, 2022)—explicitly accounts for laws which would be prior to others in such strong senses, whether by being meta-laws or by having a stricter modal status. In that respect, both approaches pose a challenge for traditional theories of the laws of nature, which is explicitly formulated in Lange (2007) and less explicitly also in the non-absolutist literature (perhaps most pertinently in the form of a challenge to accommodate scientific explanations involving counternomic, but still possible scenarios raised by Hendry and Rowbottom (2009): If the natural sciences suggest that there are meta-laws, or that some laws are necessary in a stricter sense than others, then any adequate theory of laws of nature should be able to accommodate these special statuses.

In this context, our paper is an attempt at getting clearer on what these new trends amount to, in particular by addressing the following two questions: First, what is the relation between them? (Beyond elevating some laws above the other, are they similar in any crucial respects—or indeed just two labels for the exact same idea? Or, are they, on the contrary, completely distinct and independent approaches?) Second, which approach looks more promising—and in particular, which one (if any) finds more support in scientific theory and practise? By answering these questions, we also clarify the status of the challenges the two approaches pose for theories of laws. If, as we will argue in Sect. 5, physics lends more support to the non-absolutist distinction between less and more strictly necessary laws than it does to the distinction between laws and meta-laws, then it is comparably more important for a theory of the laws to account for the former, rather than the latter distinction. If one treats adequacy as an absolute matter, one might even argue that in this case, a theory of the laws of nature which accounts for the former, but not the latter distinction can still count as adequate, whereas one which accounts for the latter, but nor the former, does not. Our paper hence contributes to addressing an important meta-philosophical question concerning the discussion about laws of nature, namely what the adequacy conditions of such theories are.

In the next three sections, we address the first question. We first introduce both approaches (Sect. 2), then show that in the specific context of Lange’s theory of laws, meta-laws coincide with more necessary laws (Sect. 3), but finally argue that the two approaches are largely distinct and independent from each other in general (Sect. 4)—calling for a comparative assessment. In Sect. 5, we compare the two approaches with respect to their applicability to physical laws, addressing the second question. We argue that physical theory and practise (at least under a suitable interpretation) are not clearly supportive of a distinction between laws and meta-laws. Section 6 contains a brief conclusion in which we compare the relative merits of the challenges that the two new approaches pose for theories of laws of nature in general in light of the previous sections.

## Two Ways of Elevating Some Laws Above Others

The first main way to elevate some laws above others is to distinguish between ’ordinary’, first-order laws, which apply to (non-nomic) natural facts, on the one hand, and meta-laws, i.e. laws of laws, on the other. According to Lange (2007), prime examples of meta-laws are variational symmetry laws, that is, invariance statements of the laws of classical physics when expressed in an action formalism. Such symmetry transformations are taken by Lange (p. 460) to explain corresponding conservation laws in classical physics in a way which is similar to how e.g. conservation laws themselves explain facts about systems described by them. In general, for Lange, meta-laws are requirements on regular first-order laws in a way analogous to the way in which regular first-order laws are requirements on facts—rather than mere byproducts of the fact that these laws hold. (See Lange, 2007, Sect. 3.)

The second main approach to elevating some laws above others is to argue that the former are necessary in a stricter sense than the latter. A standard example of a physical law which is necessary in a stricter sense, according to non-absolutist accounts, is the Pauli Exclusion Principle (*PEP*)—the statement that no two fermions in a closed system can occupy the same quantum state at once. Different accounts provide different explanations of why this law has this stricter modal status. According to Tahko’s hybrid view, the PEP is metaphysically necessary (not just nomically necessary), since it describes part of the nature of the natural kind *fermion*. (See Tahko, 2015, p. 524.) Similarly, Hirèche et al. (2021b) suggest that the PEP may be taken to be metaphysically necessary since it describes part of the nature of closed systems with two fermions, which is indicated by the fact that it is a kinematical (as opposed to dynamical) law in theories featuring fermions. While these first two accounts thus both rely on essence in order to classify some laws as metaphysically (rather than merely nomically) necessary, the non-absolutist account proposed in Hendry and Rowbottom (2009) relies on a view of properties as bundles of world-relativized dispositional profiles, classifying a law as metaphysically necessary in virtue of the relevant property’s having a constant dispositional profile with respect to all possible worlds.

How are these two approaches related? It may seem, on the one hand, that their only similarity is that they divide the laws of nature into two groups, where the members of one group are in a relevant sense prior to that of the other. Indeed, the criteria for sorting the laws into these groups seem to latch onto very different and independent features of the laws, namely what they are about, i.e. their subject matter, and their modal status, respectively. Prima facie, that a law stands in the same sort of relation to other laws in which regular laws stand to particular facts about the world tells us nothing about its modal status; likewise, that a law is metaphysically necessary does not seem to indicate any special explanatory relation between it and other laws of nature.

However, on the other hand, the two distinctions may also seem to have some substantial similarities. On Lange’s view, as we will see (Sect. 3), laws of nature are characterized by their counterfactual stability, which gives them their specific modal status (natural necessity); moreover, within the laws, Lange distinguishes various subcategories, including the subcategory of meta-laws, and each subcategory mainly distinguishes itself by being associated with a certain degree of counterfactual stability, and thereby a certain degree of necessity (cf. Lange, 2009).

Likewise, in their (abductive) argument for the metaphysical necessity of certain laws of nature, Hirèche et al’s (2021b) rely on an interpretation of Curiel’s (2016) characterization of the distinction between kinematical and dynamical constraints, according to which kinematical constraints ‘are needed to minimally pick out the systems of interest to be described by the theory *before* and *in virtue of which* dynamical evolutions can be set up.’ (Hirèche et al., 2021b, Sect. 3.2.) This suggests a constitutive priority of the kinematical laws over the dynamical laws, which they account for in terms of classifying the former laws as metaphysically and the latter laws as ‘merely’ nomically necessary. This talk of constitutive priority may very well remind one of how Lange talks about meta-laws on his own account.

Thus, at first sight, impressions on whether the two approaches are really distinct and independent may go both ways. In order to make progress, it will be particularly instructive to look at Lange’s account of laws in some more detail. First, Lange’s account is the main account of meta-laws developed in the literature.^1^ Second, it provides an ideal context to clarify what the two approaches really have in common, since it integrates not only a distinction between laws and meta-laws, but also one between less and more strictly necessary laws. We will argue that some of the apparent similarities between the two approaches are specific to Lange’s account, and that, when taking a step back from it, the two approaches are in fact largely distinct and independent from each other.

## Lange’s Account of Laws and Meta-Laws

Lange’s (2007) account of laws and meta-laws is based on the notion of counterfactual stability. Let *S* be the set of all true propositions which are not facts about laws—i.e. the set of all sub-nomic facts. A non-empty set $T\subset S$ (including all its sub-nomic logical consequences) is *sub-nomically stable* if, for any member $p\in T$ and any sub-nomic counterfactual supposition $q\in S$ logically consistent with *T*, if it had been the case that *q*, then it would still have been the case that *p* (i.e. the counterfactual $q \mathrel {\mathop \Box }\mathrel {\hspace{-1.38885pt}}\rightarrow p$ holds). Lange argues that the set $\Lambda \subset S$ formed by the laws of nature and their logical consequences (*plus* what he calls the ‘broadly logical’ truths, including not only the strictly logical truths, but also e.g. metaphysical, conceptual and mathematical ones) is sub-nomically stable. More precisely, $\Lambda$ is the *largest* sub-nomically stable set which is not maximal (i.e. which is not just *S* itself). Thus, in particular, $\Lambda$ possesses a higher grade of counterfactual stability than *S*, and indeed just the first grade of counterfactual stability above that of *S*. For Lange, it is in virtue of having that specific grade of counterfactual stability that $\Lambda$ can be said to possess a corresponding grade of necessity—namely nomic, or natural, necessity.

Having accounted for this first grade of necessity, possessed by all laws, Lange can account, in an analogous way, for higher grades of necessity, each corresponding to a non-maximal sub-nomically stable set which is strictly included in the set of laws, $\Lambda$ (Lange (2007, p. 473) shows that such stable sets are strictly ordered). One of those sets is *B*, the set of broadly logical (e.g. conceptual) truths. However, more interestingly for our present purposes, Lange (2007, pp. 473–4) argues that there is a sub-nomically stable set which is strictly included in $\Lambda$, while strictly including *B*. This set, $\Phi$, includes only part of the laws, namely the fundamental dynamical law, as well as the conservation laws which are *requirements* on the force laws; but it excludes the force laws themselves, and the conservation laws which are mere by-products of other laws. Note that $\Phi$’s being subnomically stable requires, in particular, that the conservation laws as requirements would still have held had the force laws been different—in contrast with those conservation laws which are mere by-products of the particular forces there are.

On Lange’s view, then, $\Phi$ has a higher grade of necessity than the set of all the laws, $\Lambda$, because the range of counterfactual suppositions under which the members of $\Phi$ would still have held (in connection with $\Phi$’s subnomic stability) is broader than the range of counterfactual suppositions under which the members of $\Lambda$ would still have held (in connection with $\Lambda$’s subnomic stability).

Based on this characterization of the laws and their modal force(s) in terms of subnomic stability, Lange proposes an analogous characterization of meta-laws and their modal force, in terms of what he calls *nomic* stability. He first defines the set of nomic facts, *N*, as containing all facts which are either sub-nomic or describe laws about sub-nomic facts (e.g. propositions of the form “It is a law that p”, where p is a sub-nomic fact expressing a law). Nomic stability is then defined in a way that parallels his definition of sub-nomic stability: a set $N'\subset N$ (including all its nomic logical consequences) is *nomically stable* if, for any member $p\in N'$ and any nomic counterfactual supposition $q\in N$ logically consistent with $N'$, if it had been the case that *q*, then it would still have been the case that *p* (i.e. the counterfactual $q \mathrel {\mathop \Box }\mathrel {\hspace{-1.38885pt}}\rightarrow p$ holds). While the laws in general, namely $\Lambda$, do not form a nomically stable set, the set $\Lambda _+$, containing all facts about which sub-nomic facts are laws or not, has nomic stability. Importantly, Lange argues that $\Lambda _+$ strictly contains an even more ‘exclusive’ nomically stable set, which is the set of meta-laws, $\Lambda _{meta}$; in the context of classical mechanics, the set contains ‘various symmetry principles (and perhaps certain other nomic facts as well) but [does] without the force laws, the fundamental dynamical law, the conservation laws, and other such first-order laws’ (Lange, 2009, pp. 115–6).

It should be noted that, according to Lange, not all symmetries are meta-laws: only those symmetries which are requirements on certain laws, as opposed to being mere by-products of them, qualify as meta-laws. The former sort of ‘symmetry principles would still have held had the fundamental dynamical laws been different, or had the force laws governing the actual kinds of forces been different, or had there been an additional kind of force besides the actual kinds...Some symmetry principles may be meta-laws while others hold as byproducts of the ordinary laws-“by chance”...’ (Lange, 2007, p. 475).

On Lange’s view, the grade of necessity associated with $\Lambda _{meta}$, the set of metalaws, including symmetries as requirements, is even higher than the grade of necessity associated with $\Phi$, the subset of the laws which contains the fundamental dynamical law and the conservation laws as requirements (but leaves out the force laws and the conservation laws as mere by-products). This is because the range of counterfactual suppositions under which the metalaws would still have held (in connection with $\Lambda _{meta}$’s *nomic* stability) is broader than the range under which the relevant subset of the laws, including conservation laws as requirements, would still have held (in connection with $\Phi$’s *subnomic* stability). In particular, symmetries as requirements have a higher grade of necessity than conservation laws as requirements. And this higher degree of necessity, according to Lange (2007, p. 478), is the reason why the relevant symmetries can explain the corresponding conservation laws, but not the other way around.

Importantly, $\Lambda _{meta}$ has a higher grade of necessity than both $\Phi$ and, a fortiori, $\Lambda$. Thus, on Lange’s account, meta-laws are necessary in a stronger sense than ordinary, non-meta laws.

## Meta-Laws and More Necessary Laws: Comparing and Contrasting the Two Approaches

Let us now consider how meta-laws and more strictly necessary laws are related in the particular context of Lange’s account—then, we will discuss how the two are related more generally.

As noted earlier, Lange’s account of laws and meta-laws is at the same time a non-absolutist account, since it attributes different modal statuses to different categories of (meta-)laws. One difference with the non-absolutist views mentioned earlier (Hendry & Rowbottom, 2009; Tahko, 2015; and Hirèche et al., 2021a, 2021b) is that those views draw a line between laws that are *metaphysically* necessary and laws that are merely naturally necessary, while Lange distinguishes grades of modal forces *within* natural necessity. Another difference is that, while other non-absolutist accounts distinguish exactly two modal statuses within laws, Lange’s account has (at least) three different grades of necessity among laws (in the broad sense, including meta- and non-meta-laws): there is, first, the necessity of $\Lambda$, which is the weakest form of necessity which laws may possess on his account; second, the stronger necessity of $\Phi$; third, the necessity of $\Lambda _{meta}$, which is the strongest. Still, since Lange’s account also attributes different modal statuses to different laws, in contrast with the main traditional theories of laws, there is a clear sense in which it counts as a non-absolutist account.^2^

It should be stressed that Lange’s account is not simply an account of meta-laws which also somehow happens to be a non-absolutist account. Indeed, Lange draws the line between meta-laws and laws exactly where he draws the line between those laws which have the highest grade of (natural) necessity and those laws which have a lesser grade of necessity: as we have seen, the set of meta-laws, $\Lambda _{meta}$, possesses a modal force which is higher than that of all other, non-meta laws. This establishes a clear connection between meta-laws and more necessary laws on Lange’s account: being a meta-law directly implies being a more necessary law.

Conversely, does being a more necessary law imply being a meta-law? It depends what exactly we mean by “more necessary”. As we have seen, on Lange’s view, laws may have (at least) three types of modal force. On the one hand, it is not the case that having *any* form of necessity which is stronger than that of $\Lambda$ implies being a meta-law—a law can be a member of $\Phi$, and thereby possess such a stronger necessity, while not being a meta-law. However, on the other hand, it is still the case that, on Lange’s view, having the *strongest* form of nomic necessity does directly imply being a meta-law.

Thus, there is a very close connection between being a meta-law and having a stronger (the strongest) necessity on Lange’s account. Two questions now arise: First, is this close connection on Lange’s account a mere coincidence, or is there a deeper reason for it? Second, is there also such a close connection between the meta-law approach and the modal approach *in general*, even outside his account?

In order to answer these questions, it will be useful to say a bit more about meta-laws themselves. Lange takes it as a major advantage of his account of laws that it is able to account, not only for first-order laws, but also for meta-laws. Accordingly, he presents a challenge to rival views, in particular the main traditional theories of laws: ‘Philosophical analyses of natural law should be asked to account not only for the distinction between first-order laws and accidents, but also for the distinction between meta-laws and byproducts of the ordinary laws.’ (Lange, 2007, p. 479)

Notably, as Lange (2007, p. 479) suggests, and as Yudell (2013) argues, that challenge should naturally be ‘account-specific’: to make sense of what a meta-law of nature is, we first have to make sense of what laws of nature proper are—which of course is a comprehensive debate of its own. If meta-laws are laws of laws, being related to laws in the same way as laws are related to mere sub-nomic facts, then whether and how a given theory of the laws is able to meet Lange’s challenge of accounting for meta-laws should be dependent on how, in the first place, laws are related to the sub-nomic facts they govern *according to that specific theory*.

Clearly, that latter relation may be, and actually is, characterized in vastly different ways by different theories of laws of nature. Think for example of the DTA-theory on the one hand, according to which the laws govern the facts, and the best-systems analysis on the other, according to which the laws are particular generalizations which derive from the facts. The main insight of Yudell (2013) is that this theory-relativity also pertains to the relation between meta-laws and laws, which is after all supposed to parallel the relation between first-order laws and facts. Accordingly, Yudell fleshes out the relation between laws and meta-laws by first pinpointing theoretical roles played by regular laws according to a particular account of laws (Lange’s counterfactual stability account, Humeanism^3^ or DTA in Yudell (2013)) with respect to the sub-nomic facts, and then characterizing the meta-laws as those laws which play the same roles not with respect to these facts, but to the regular, i.e. non-meta laws of nature.

In the case of Lange’s account, Yudell identifies four such roles (see Yudell, 2013, p. 350). First, laws explain sub-nomic facts, i.e. facts which are not explicitly about lawhood. Second, they support counterfactual conditionals about sub-nomic facts. Third and fourth, ‘laws are more necessary than accidental sub-nomic facts, and laws are inductively confirmed by sub-nomic facts.’ (ibid.) Yudell argues that meta-laws indeed play these roles with respect to first-order laws on Lange’s account.

For our present purposes, what is particularly interesting about Yudell’s characterization of meta-laws in terms of roles is how it helps us see that the very close connection that we pointed out earlier between meta-laws and more necessary laws on Lange’s account is in fact no coincidence; rather, it largely follows from the fact that, ultimately, the roles which laws are supposed to play on his account, and therefore the analogous roles which meta-laws are supposed to play with respect to first-order laws, are largely *modal* in character.

Most strikingly, one of the roles played by laws with respect to the sub-nomic facts they govern, on Lange’s account, is indeed to be *more necessary* than those facts. Clearly, as we have seen, meta-laws on Lange’s account play that same role with respect to first-order laws. Thus, it is no coincidence that meta-laws are also more necessary laws on Lange’s account: being more necessary is one of the roles they have to play as meta-laws; it is part of what makes them meta-laws.

Likewise, consider the first role which Yudell identifies for meta-laws on Lange’s account: their explanatory role with respect to first-order laws. In his paper on meta-laws, Lange’s focus is on the example of symmetry principles which constitute requirements on first-order laws. They, Lange writes, ‘explain why conservation laws hold because symmetry principles are laws of laws–meta-laws governing (and helping to explain) ordinary (i.e., non-meta, ‘first-order’) laws in a manner precisely analogous to the way in which those laws govern (and help to explain) ordinary facts and events.’ (Lange (2007), p. 458.) Lange ultimately explains the notion of explanation (and also the notion of governance, which he seems to identify in this context) at play here in modal terms: ‘The laws’s explanatory power derives from their necessity; [...] I shall take this account and apply it one level higher in order to understand what it would be for symmetry principles to be meta-laws and hence to possess a species of necessity stronger than conservation laws possess, making symmetry principles explanatorily prior to conservation laws.’ (Lange (2007), p. 458.) Thus, just like the role of being more necessary, the role of being explanatory is also clearly, though indirectly, modal in character: on Lange’s account, meta-laws are meta-laws partly because they explain first-order laws, but this, in turn, is the case because meta-laws are more necessary than first-order laws.

According to Yudell, on Lange’s account, meta-laws also support counterfactual conditionals about first-order laws—just like first-order laws themselves support counterfactual conditionals about sub-nomic facts. As we have seen (§2.2), it is clear that meta-laws on Lange’s account indeed play this role with respect to other (first-order) laws. And this role is also quite clearly modal in character—it basically follows from meta-laws possessing a stronger form of necessity, which on Lange’s account amounts to their possessing a stronger form of counterfactual stability.

It might be less clear that the fourth and last role identified by Yudell for meta-laws on Lange’s account, namely that of being inductively confirmed by nomic facts (in the same way as first-order laws are inductively confirmed by sub-nomic facts) also has a modal character. Be that as it may, the fact that at least three of the four main roles which characterize meta-laws on Lange’s account are clearly modal already gives us some strong reasons to think that the very close link between meta-laws and more necessary laws is a core feature of his account, rather than a mere coincidence.

What does this tell us about Lange’s challenge? Briefly put, that with respect to Lange’s theory, meeting the challenge amounts to making room for a class of laws which is necessary in a stricter sense than regular laws of nature.

Let us now take a step back from Lange’s account and discuss how the two approaches to elevating some laws above others are related more generally. Given Yudell’s insight that the relation between meta-laws and laws is specific to which theory of the laws of nature one adopts, this discussion should also be theory-specific. We will not be able to discuss all such theories, but to make our main point, this is in fact not necessary. Our point is that the close connection between the two approaches is a specific feature of Lange’s theory of the laws of nature: In general, being a meta-law does not imply being a more necessary law, and being a more necessary law does not imply being a meta-law.

To illustrate the point that the first implication does not hold in general, we will consider one of the main traditional theories of laws, namely the DTA theory (cf. Dretske, 1977; Tooley, 1977; Armstrong, 1983). For the sake of simplicity, we will focus on Armstrong’s version of the theory.

The core idea of this version of the theory is that a law of nature is the obtaining state of affairs that certain universals stand in the relation of necessitation. Using a notational schema to capture this idea, the law that all *F*s are *G*s consists in the obtaining state of affairs *N(F,G)*.

Yudell argues that Armstrong’s theory, unlike the regularity theory, can indeed account for meta-laws. As in case of Lange’s theory, Yudell identifies four roles which laws play with respect to the natural facts in order to characterize the analogous relation between meta-laws and laws. Mentioning only the versions of the roles for meta-laws, these roles are (cf. Yudell, 2013, pp. 360ff): (i) meta-laws are nomically necessary, (ii) they explain first-order laws, (iii) they are inductively confirmed by the first-order laws they are about, and (iv) they support counterlegals, i.e. counterfactual conditionals about the first-order laws.

The question we are interested in here is of course whether a law which fits this characterization of a meta-law is also more strictly necessary than a first-order law according to the DTA-theory. Yudell’s development of a DTA-based account of meta-laws makes clear that this is not the case. According to Yudell, DTA-meta-laws are states of affairs of the form *N(N,P)*, i.e. states of affairs to the effect that the necessitation relation applies to itself and a higher-order universal *P* which holds between the universals involved in a first-order law. Crucially, meta-laws hence involve the same necessitation relation as first-order laws. This means that, unlike in Lange’s theory, they are not more strictly necessary than first-order laws, but rather necessary in the exact same sense,^4^

To be fair, it is not entirely clear how the standard DTA-theory could even accommodate a distinction between more and less strictly necessary laws. Hirèche et al.’s (2021b) effort to develop a non-absolutist version of the DTA-theory shows that substantial additions to the theory might be needed to accommodate this distinction. Be that as it may, the preceding discussion provides a clear counterpoint to the claim that meta-laws are in general more strictly necessary than first-order laws.

Conversely, outside of Lange’s specific account, it does not seem that being a more necessary law implies being a meta-law in general. This is already suggested by the fact that none of the proponents of the non-absolutist accounts (Hendry & Rowbottom, 2009; Tahko, 2015; and Hirèche et al., 2021a, 2021b) are arguing, or even indicating, that the more necessary laws on their accounts are also meta-laws. To briefly illustrate, on Tahko (2015)’s account, the more necessary laws distinguish themselves by finding their source in the essence of some natural kind; and this does not imply, or even suggest, that they should be regarded as meta-laws.^5^

To sum up, the striking similarity between the meta-law approach and the non-absolutist approach on Lange’s specific account does not generalize: the two approaches are largely distinct and independent from each other.

What does this tell us about Lange’s challenge? Since being a meta-law and being a more strictly necessary law do not generally amount to the same thing, Lange’s challenge can not in general be met by making room for a class of laws which are more necessary than ordinary laws. One might even argue that this shows Lange’s challenge to be somewhat ambiguous: We can think of the challenge as concerned with the pre-theoretical notion of a meta-law, just as Yudell did, or we could rather focus on Lange’s modal explication of what it takes to be a meta-law within his theory. The latter option of course just turns Lange’s challenge into the challenge which non-absolutist theories pose for traditional theories of the laws of nature, that of making room for a special class of more strictly necessary laws.

Like Lange’s challenge, which is based on the observation that a number of physicists appear to de facto treat symmetry laws as meta-laws, this ‘non-absolutist’ challenge is motivated by physics. Tahko for example points out that the variance of the fine structure constant over time^6^ is a live hypothesis investigated by experimental physicists (cf. Tahko, 2015, p. 520), which strongly suggests that laws involving it, such as for example the derivative law of the velocity of the electron (relative to the velocity of light) in the first orbit of Bohr’s atom model, are less strictly necessary than laws which do not. It seems that a theory of the laws of nature which aims for naturalistic adequacy should take such arguments into account and should hence address the challenge to accommodate laws with different modal statuses.

Since motivations for both approaches have been provided by their respective proponents (see Lange, 2007; Hendry & Rowbottom, 2009; Tahko, 2015; Hirèche et al., 2021b), it appears that, at least at first sight, theories of the laws of nature have to face two distinct challenges. But are both challenges equally pressing? If it could be shown that one of the two underlying distinctions between different kinds of laws was ill-motivated, then this could undermine the claim the associated challenge has to serve as a test for the adequacy of a general theory of the laws of nature. Is there any reason to think that one of the two approaches is less well-motivated than the other? In the next section, we address this question focusing on the applicability of the two approaches to physical laws, in the light of physical theory and practise.

## Meta-Laws and More Necessary Laws from the Perspective of Physics

In this section, we turn to physics: given the presupposition that physical laws have modal force, a presupposition common to both the modal and the meta-laws approach, what support do our best physical theories provide for the ideas of non-absolutist law accounts—and what support for meta-laws? We begin with the case for non-absolutist law accounts. Standardly, theories of the laws of nature are absolutist, meaning they presuppose that all laws have the same modal status. However, in the recent literature, Tahko (2015), Hendry and Rowbottom (2009) as well as Hirèche et al. (2021a; b) (partly in response to Tahko) have all made a case that physics suggests that there are laws of different modal status. We cannot review all of these points here but would like to focus on what we take to be the strongest case from physical practice for a non-absolutist take on laws—the kinematical/dynamical distinction.

According to the kinematical/dynamical distinction as conceptualised by Curiel (2016), statements of a physical theory are either fundamentally constitutive of systems (kinematical) or further ascriptions to such systems (say in the forms of laws, properties, etc. in whatever precise sense). The distinction has been explicated by Curiel through a syntactic criterion: statements that are explicitly expressible through accepted basic variables are kinematical—all other statements, that is, statements which involve placeholders, are dynamical. Take Maxwell’s electrodynamics with basic variables ${\textbf {E, B}}$ and their derivatives. Then $\nabla {\textbf {B}} = 0$ which just involves the basic variable ${\textbf {B}}$ counts as kinematical. In contrast, statements that require recourse to placeholder objects that are just stand-ins for structure that can differ from model to model—such as the charge density $\rho$ in the Maxwell equation $\nabla {\textbf {E}} = \rho$—are dynamical.^7^

The kinematical/dynamical distinction in this form applies to all physical theories there are, i.e., is ominipresent across modern physics (see appendix in Hirèche et al. (2021b) for a demonstration of its omnipresence in spacetime theories). Assuming a realist approach to metaphysics, it is then natural to re-read the kinematical/dynamical distinction as one between two kinds of necessity: kinematical constraints, describing the very nature or essence of the physical entities or systems involved, are metaphysically necessary, while dynamical laws are ’merely’ nomically necessary (Hirèche et al., 2021a, p. 10).^8^ As already mentioned, the claim that current scientific theory and practise suggest that some laws are more necessary than others has also been defended in other ways by other proponents of non-absolutist accounts of the laws—whether these non-absolutist accounts are at the same time meta-laws accounts (Lange, 2007) or not (Tahko, 2015; Hendry & Rowbottom, 2009).

What about meta-laws? Are they also naturally suggested by a realist reading of physics? We will argue that this is more questionable than usually thought.

Building on a tradition from physics of reading symmetry laws as directly concerning laws that at least dates back to Wigner, Lange (2007) takes it that symmetry principles are meta-laws in that they concern laws in a way somewhat analogous to how laws concern facts:

the regularity associated with a symmetry principle ...concerns laws; it is a regularity in the regularities associated with laws governing non-nomic facts. ...It is “meta” to the “ordinary laws”—the first-order laws, the laws governing the non-nomic facts (i.e., laws of the form “It is a law that m” where m is non-nomic). (p. 474)

Now, it is vastly uncontroversial that symmetry principles do explicitly concern laws. As Brading et al. (2021) summarise succinctly in their authoritative survey article on how symmetries occur in physical practice:

we may attribute specific symmetry properties to phenomena or to laws (symmetry principles). It is the application with respect to laws, rather than to objects or phenomena, that has become central to modern physics (Sect. 1)

However, the fact that symmetries explicitly concern laws does not straightforwardly, at least not without further argument, make them *govern* laws yet—and even if so, not yet necessarily in the way that laws govern non-nomic facts.^9^ A decisive question is then whether symmetry principles really only concern laws or not facts in a significant sense as well.

To make progress with the question, it is important to distinguish more carefully between the various senses in which symmetry principles (‘symmetry laws’) concern laws in physics: most centrally, there are dynamical symmetry principles (i.e., statements that the equations of motion are invariant under a certain transformation),^10^ and variational symmetry principles (i.e., statements that the action is invariant^11^). In the remainder of the section we will argue that physics neither clearly supports the view that symmetry principles govern laws in the same manner as those laws govern natural facts in case of dynamical, nor in case of variational symmetries. In case of dynamical symmetries, we show that whether symmetries concern other laws in any substantial sense heavily depends on the formulation of the underlying physical theory. In case of variational symmetries, we argue that Lange’s argument for them being meta-laws overlooks an important consequence of Noether’s theorem, namely that conservation laws, laws which are not meta-laws according to Lange, concern other laws in the same manner as variational symmetry principles do.

A sense in which dynamical symmetry laws (i.e., referring to the equations/laws of motion) are taken to concern laws in physical practice is captured by Baker’s (2010) formalisation of the functional role of dynamical symmetry laws as one finds it in Hamiltonian classical mechanics and canonical quantum mechanics:

Symmetries are then given by transformations that leave the dynamics (diachronic laws) unchanged. Mathematically, this means they must commute with the dynamics [represented by *U*], so that $U(t') T = T U(t')$ for every symmetry transformation *T* and every time $t'$. (p. 1158)

Notably, both dynamical (diachronic) laws and dynamical symmetry laws are represented as transformations on physical states by such a formalism. The fact that symmetry laws are transformations that stand in a commutation relation to diachronic laws, allows for a view of symmetry laws as requirements on (other) laws qua *laws*. It is not clear, however, how—upon such a formulation—symmetry laws can ever be sensibly said to govern diachronic laws analogous to how diachronic laws are said to govern facts: while symmetry laws literally put a commutation constraint on other laws, they are still on par with diachronic laws in that they both act on states. (If symmetry transformations just acted on laws, one could simply not account for the commutation relation between symmetry laws and diachronic laws.)^12^

Admittedly, dynamical symmetry laws can be rendered as meta-laws again when departing from Baker’s formulation of the relationship between *U* and *T* by rewriting the commutation relation between the transformations *U* and *T* as $U = T^{-1} U T$. This alternative formulation of the same physics suggests thinking of symmetry transformations as transformations of dynamical transformations—and thus of symmetry laws as meta-laws after all.

Furthermore, one might object that physics formulated in an explicitly four-dimensionalist manner does away with the notion of (evolving) states—and that on such formulations, symmetry principles indeed concern only laws then.^13^

The upshot so far then is that, whether or not dynamical symmetry laws can even be read as meta-laws, turns out to be formulation-dependent. However, as pointed out earlier in this section, even if symmetry laws just concern laws (as is arguably the case on a four-dimensionalist formulation), it would not automatically follow that they *govern* laws in the same way in which laws govern facts.

The other decisive class of symmetry laws is arguably that of variational symmetries, i.e., transformations that leave the action invariant (or, equivalently, its integrand—the Lagrangian–invariant up to a boundary term).^14^ Note that Brading (2002); Brading and Brown (2003) have argued repeatedly that the physics is in the dynamics while an action formalism solely amounts to mathematical auxiliary structure, consequently motivating a dismissal of the latter’s metaphysical relevance. On the other hand, one might very well want to give ontological relevance to more than the dynamical equations of motion and related structure—for the (classical) action, it might in particular be said that it contains information about the limit from the quantum. In any case, we simply accept for the moment that variational symmetries are metaphysically relevant.

Now, variational symmetries, on the one hand, and conservation laws, on the other hand, stand in a one-to-one correspondence through the Noether formalism.^15^ While Lange accepts conservation laws as natural laws that put requirements on other natural laws, he apparently would like to render variational symmetries as meta-laws. In fact, Lange seems to consider it a strength of his account that, despite the extensional equivalence displayed in the Noether approach to symmetries and conservation laws, he can provide a sense in which variational symmetries qua requirements are more robust than conservation laws qua requirements. In doing so, he misreads—or so we will show in a moment—the conservation law side of the Noether correlation as independent of laws while solely attributing such a dependence to symmetry laws; this misreading may then invite giving conservation laws a different status from symmetries.^16^

Let us demonstrate the point by considering the simple Noetherian case in which the invariance of an action *S* under time-translation corresponds to the conservation of a non-trivial quantity *E*, *all provided that* relevant equations of motion—derived from *S* via Hamilton’s principle—hold. Turning the conservation law into a general requirement on actions (and in this sense on laws), amounts to requiring that

*(Noether energy conservation)* For all *S* fulfilling Hamilton’s principle and of form $S := \int L dt$: there is $E := \left( \frac{\partial L}{\partial \dot{x}} \dot{x} - L \right) \ne 0$ with $dE/dt \overset{on-shell}{=}\ 0$.

where ‘on-shell’ clarifies that the equality only holds in virtue of the fact that Hamilton’s principle applies (i.e., the equations of motion hold).

In contrast, a conservation law qua requirement à la Lange would, in this context, take the form ‘There is a (non-trivial) quantity *E:* = … with $dE/dt = 0$.’^17^ But such an characterisation of conservation law does not faithfully capture what is meant by a conservation law in the Noether context, i.e., *(Noether energy conservation)* above.

So, it is not only that variational symmetry laws are explicit requirements on the action (and in this sense on laws)^18^—as Lange would like to have it—but so are conservation laws: the conserved quantities involved in a Noetherian conservation law are simply undefined if not defined in reference to the Lagrangian and thus to *S*. There is thus no difference between variational symmetry laws and conservation laws as to whether they are explicitly statements about laws.

Given that one adopts Lange’s mindset of looking for indications for there being meta-laws in physical theory, we take it that a law’s being an explicit statement about another law should be considered a good indicator for that law’s being a meta-law. Our argument can hence be taken to show that, independently of which philosophical analysis of meta-lawhood one settles for, if one considers variational symmetry laws to be meta-laws, then one should also be prepared to assign the same status to (Noetherian) conservation laws. Since Lange explicitly argues that given his theory, symmetries which express requirements are, and conservation laws are not meta-laws (cf. Lange, 2007, Sect. 5), his theory is at odds with the simple physics-based criterion for being a meta-law which we relied on in our argument. One may hence take our argument to question Lange’s criterion for a law’s being a meta-law, but also more generally, Lange’s initial assumption that there is a robust distinction between laws and meta-laws inherent in physics.

The preceding arguments question the legitimacy of Lange’s challenge and, if non-absolutist like Hendry and Rowbottom, Tahko, and in particular Hirèche et al., who have provided detailed arguments to the conclusion that physics suggests non-absolutism, are right, tips the balance towards non-absolutism and the associated challenge that adequate theories of laws of nature should account for a modal distinction between laws which are less and more strictly necessary.

## Conclusion

We clarified the relations between the two main emerging approaches to elevating some laws of nature above others, the meta-laws and the modal approach, showing how they are generally distinct and independent projects. With the situation thus clarified, we went on to critically consider the motivations for each approach. On the one hand, the idea that current physical theory and practise suggest different modal forces for different laws of nature finds substantial support in the recent work of various authors, including not only the main defenders of non-absolutism but also the main proponent of meta-laws. On the other hand, support for the (additional) claim that there are meta-laws beyond ordinary laws seems weaker in comparison. In particular, we argued that even symmetries which explicitly concern other laws may amount to requirements on these laws, but do not straightforwardly govern them in the way laws govern particular facts.

On those bases, we conclude that physical theory and practice appear to be more directly supportive of the distinction between less and more strictly necessary laws than of the (additional) distinction between ordinary laws and meta-laws. If so, the challenge posed by non-absolutism for traditional theories of the laws of nature is more pressing than Lange’s challenge to account for meta-laws.

## Footnotes

1 The only other works in this direction are Yudell (2013) and Friend (2016). These papers directly connect to Lange’s account. Hicks (2019) provides a critical response.

2 Indeed, Hirèche et al. (2021a) specifically suggest that, in principle, a non-absolutist may hold that no law is metaphysically necessary, and rely instead on the different grades of nomic necessity at work in Lange’s account. But note that the necessities of $\Lambda$ and $\Phi$ are related to one another by restriction (in the sense of Fine, 2002), whereas $\Lambda _{meta}$ is not. Such a non-absolutist account would thus have to exclude $\Lambda _{meta}$.

3 For more on meta-laws and Humeanism, see also Shi (In preparation).

4 Cf. Yudell (2013), p. 363. To briefly address the other roles: Meta-laws are nomically necessary, since they are necessitating states of affairs; they explain first-order laws in virtue of their nomological necessity and they are inductively confirmed by the laws they govern in a way analogous to how first-order laws are confirmed by the facts they govern according to the DTA-view. The fourth role is a bit more problematic, but Yudell proposes a way to accommodate it, with the caveat that it may not be to the liking of orthodox defenders of the DTA-theory. See Yudell (2013, pp. 364–6).

5 The implication likewise fails to hold for the metaphysical criteria for being a more strictly necessary law set out by Hirèche et al. (2021b) (involving essentially related universals) and by Hendry and Rowbottom (2009) (stability of the dispositional profile of the dispositional property involved in the law throughout all possible worlds).

6 Note that the interest is here on the variance over time. It is rather uncontroversial that, in the more elaborate context of quantum electrodynamics, the value of the (measurable) fine structure’s constant depends on the probing energy.

7 It is a delicate question whether natural constants should count as placeholder objects or rather fixed objects. Consider the theory of Newtonian gravitation for two particles which has the equation of motion $\ddot{x} = G \frac{m_2}{r^2}$ for a particle of mass $m_1$ relative to a particle of mass $m_2$. Then the sense in which $m_2$ is seen as a placeholder, namely as a variable mass, seems strikingly different from the sense in which the natural constant *G* could be seen as a placeholder. Standardly, in Newtonian gravity, *G* is seen as fixed across all models. Generally, we will understand natural constants as kinematical in the following; we can thus do without explicitly mentioning natural constants.

8 Even though the argument of Hirèche et al. from the kinematical/dynamical distinction comprises several steps, from the third step on, these only involve commitment to widely held background assumptions whose overall conjunction is widely agreed with as well; in other words, the metaphysical assumptions at play from step three on are simply elements of the well-familiar Kripkean package deal accepted by many contemporary metaphysicians. The significant leap is arguably undertaken with re-interpreting the semantic relevance of kinematical structure in a thingly fashion: The success of a theory also involves the success of the kinematical/dynamical distinction; thus, on a consequent realist line—or so the intuition of Hirèche et al. (2021b) goes—it is natural to give the distinction an ontological status.

9 Surely, Lange has found a way with his counterfactual-based account of laws to talk of symmetries qua meta-laws as governing laws in a way directly analogous to how laws, on his account, govern non-nomic facts. But the question is of course whether such a minimalist account of governing is satisfactory. Arguably, on his account, even logical laws should count as governing physical laws—a consequence which leads the governing law intuition ad absurdum.

10 It is important to recognise that ‘dynamical’ here only narrowly refers to equations of motion and is not meant in the sense of the kinematical/dynamical distinction.

11 Notably, actions only are relevant for encoding laws once featuring in an actual physical statement, such as through Hamilton’s principle, the posit that the action gets extremal between start- and end‘point’.

12 A take on symmetries as being about first-order facts seems reminiscent of Hicks’ (2019) critical objection to Lange’s adherence to meta-laws as to that symmetries are, rather than [...] higher-order principles to which the laws must adhere, ... generalizations about the first-order facts of the world. They are justified because they encode information about the structure of spacetime (in the case of global external symmetries, those we’ve been focussing on thus far) or the property structure of the world (as, for example, the charge conjugation symmetry of classical electrodynamics does). This information provides us with some important empirical information: in addition to giving us quite general information about what properties and relations are (and aren’t) instantiated in the world, symmetry principles tell us when two isolated subsystems will behave in the same way, despite having different connections to the rest of the world. (p. 1289) Note though that there seems to be differences to Baker’s account: symmetries seem to be conceived by Hicks as posited identities between basic observables—and as such completely independent of other (dynamical) laws. Moreover, Hicks is not necessarily concerned with states at different times but arguably also with facts across time. Even if Hicks’ rendering seems like a fair option, it is definitely—as stressed before with the quote from Brading et al.—not how practitioners conceive of dynamical symmetries but rather a metaphysical re-construal thereof. It is easy to mediate between both sides here, however, as they are not contradictory: symmetries can occur both as constitutive of the basic set-up (defining its basic variables)—as Hicks would like to have it—as well as with respect to laws explicitly—as Brading et al., Baker and ourselves would like to put the emphasis, i.e., they are either kinematical or dynamical. In particular, they should thus either exclude, or include placeholder variables, and thus either leave usual laws with placeholder relations unmentioned or explicitly refer to them. It is worth noting that Lange does acknowledge a reading of symmetries in line with Hicks but goes on to discard it: On this view, spacetime symmetry principles are grounded directly in a fundamental feature of the universe and so presumably they are explanatorily prior to conservation laws. However, as we have just seen, even natural philosophers (such as Lagrange and Hamilton) who did not deny the reality of absolute space, time, and motion regarded symmetries as explanatorily prior to conservation laws. Even if there were absolute positions, times, directions, etc., the laws could nevertheless fail to privilege any. (p. 466) Arguably, there is thus some agreement between Lange and us here—contra Hicks—on that symmetries are not necessarily constitutive about space, time, charge, etc. simpliciter alone but do concern the laws explicitly.

13 An example of such physics is special or general relativistic physics in their respective covariant formulations—as opposed to their initial value formulations in a $3+1$ formalism working with hypersurface foliations (Wald, 2010, section 10).

14 Or, in the quantum/statistical context, the partition function. Counterparts to Noether’s theorem then exist in the form of what are known as Ward identities.

15 Variational symmetries imply dynamical symmetries but the converse does not necessarily hold (see Brown & Holland, 2004). Note further that variational symmetry laws (for instance, of the form that actions have to be time-translation invariant) are also dynamical statements in our terminology with respect to the ‘kinematical/dynamical’ distinction since they involve placeholder structures.

16 And again, as we argued above, the mere fact that symmetry laws explicitly concern other laws does not make them meta-laws yet.

17 Lange literally states: ‘A conservation law is associated with a regularity: that in every isolated system, a given quantity is conserved.’ (p. 466)

18 The symmetry law counterpart in the case considered is: ‘For all *S* fulfilling Hamilton’s principle and of form $S:= \int L dt$: $T[S] = S$ where *T* is time-translation.’

## References

[CR1] Armstrong, D. M. (1983). *What is a law of nature?*. Cambridge University Press.

[CR2] Baker, D. J. (2010). Symmetry and the metaphysics of physics. *Philosophy Compass,**5*(12), 1157–1166.

[CR3] Bird, A. (2007). *Nature’s metaphysics*. Oxford University Press.

[CR4] Brading, K., & Brown, H.R. (2003) Symmetries and Noether’s theorems. *Symmetries in Physics: Philosophical Reflections,* 89–109

[CR5] Brading, K., Castellani, E., & Teh, N. (2021). Symmetry and Symmetry Breaking. In E. N. Zalta (Ed.), *The Stanford encyclopedia of philosophy, fall* (2021st ed.). Stanford University. https://urldefense.com/v3/__https://plato.stanford.edu/archives/fall2021/entries/symmetrybreaking/__;!!NLFGqXoFfo8MMQ!orTF78oUy8LSG74F9ujh7EonOme0i0-VAUJcYfRa5R9faI4CnuA4UH17uiRWjxesmhUkbPlElRqQ25h1kUBVQ0ZqCTK9KeFS$

[CR6] Brading, K. A. (2002). Which symmetry? Noether, weyl, and conservation of electric charge. *Studies in History and Philosophy of Science Part B: Studies in History and Philosophy of Modern Physics,**33*(1), 3–22.

[CR7] Brown, H. R., & Holland, P. (2004). Dynamical versus variational symmetries: Understanding Noether’s first theorem. *Molecular Physics,**102*(11–12), 1133–1139.

[CR8] Carroll, J. (1994). *Laws of nature*. Cambridge University Press.

[CR9] Chen, E. K., & Goldstein, S. (2022). Governing without a fundamental direction of time: Minimal primitivism about laws of nature. In Y. Ben-Menahem (Ed.), *Rethinking the concept of law of nature* (pp. 21–64). Springer.

[CR10] Curiel, E. (2016). Kinematics, dynamics, and the structure of physical theory. arXiv preprint arXiv:1603.02999

[CR11] Dretske, F. (1977). Laws of nature. *Philosophy of Science,**44*(2), 248–268. 10.1086/288741

[CR12] Ellis, B. (2001). *Scientific essentialism*. Cambridge University Press.

[CR13] Fine, K. (2002). Varieties of necessity. In T.S. Gendler, J. Hawthorne (Eds.), *Conceivability and possibility* (pp. 253–281). Oxford Up

[CR14] Friend, T. (2016). Laws are conditionals. *European Journal for Philosophy of Science,**6*(1), 123–144. 10.1007/s13194-015-0131-z

[CR15] Hendry, R. F., & Rowbottom, D. P. (2009). Dispositional essentialism and the necessity of laws. *Analysis,**69*(4), 668–677.

[CR16] Hicks, M. T. (2019). What everyone should say about symmetries (and how humeans get to say it). *Philosophy of Science,**86*(5), 1284–1294. 10.1086/705475

[CR17] Hirèche, S., Linnemann, N., Michels, R., et al. (2021). The modal status of the laws of nature. Tahko’s hybrid view and the kinematical/dynamical distinction. *European Journal for Philosophy of Science,**11*(1), 1–15. 10.1007/s13194-020-00335-4 10.1007/s13194-020-00335-4 PMC7801292 33488848

[CR18] Hirèche, S., Linnemann, N., Michels, R., et al. (2021). The strong arm of the law - a unified account of necessary and contingent laws of nature. *Synthese,**199*, 10211–10252. 34970011 10.1007/s11229-021-03243-z PMC8668859

[CR19] Lange, M. (2007). Laws and meta-laws of nature: Conservation laws and symmetries. *Studies in History and Philosophy of Science Part B: Studies in History and Philosophy of Modern Physics,**38*(3), 457–481. 10.1016/j.shpsb.2006.08.003

[CR20] Lange, M. (2009). *Laws and lawmakers science, metaphysics, and the laws of nature*. Oxford University Press.

[CR21] Lewis, D. K. (1973). *Counterfactuals*. Blackwell.

[CR22] Maudlin, T. (2007). *The metaphysics within physics*. Oxford University Press.

[CR23] Shi, S.Y. (In preparation) Are symmetry principles meta-laws?

[CR24] Tahko, T. E. (2015). The modal status of laws: In defence of a hybrid view. *Philosophical Quarterly,**65*(260), 509–528. 10.1093/pq/pqv006

[CR25] Tooley, M. (1977). The nature of laws. *Canadian Journal of Philosophy,**7*(4), 667–98. 10.1080/00455091.1977.10716190

[CR26] Wald, R. M. (2010). *General relativity*. The University of Chicago Press.

[CR27] Yudell, Z. (2013). Lange’s challenge: Accounting for meta-laws. *British Journal for the Philosophy of Science,**64*(2), 347–369. 10.1093/bjps/axs008

